# Development and validation of machine learning-based risk prediction models of oral squamous cell carcinoma using salivary autoantibody biomarkers

**DOI:** 10.1186/s12903-022-02607-2

**Published:** 2022-11-24

**Authors:** Yi-Ju Tseng, Yi-Cheng Wang, Pei-Chun Hsueh, Chih-Ching Wu

**Affiliations:** 1 Department of Computer Science, National Yang Ming Chiao Tung University, Hsinchu, Taiwan; 2 Computational Health Informatics Program, Boston Children’s Hospital, Boston, MA USA; 3 Department of Information Management, Chang Gung University, Taoyuan, Taiwan; 4 Department of Fundamental Oncology, University of Lausanne, Lausanne, Switzerland; 5 Ludwig Institute for Cancer Research, University of Lausanne, Epalinges, Switzerland; 6 Graduate Institute of Biomedical Sciences, Chang Gung University, Taoyuan, Taiwan; 7 Department of Medical Biotechnology and Laboratory Science, College of Medicine, Chang Gung University, No. 259, Wenhua 1St Rd., Guishan Dist., Taoyuan City, 33302 Taiwan; 8 Department of Otolaryngology-Head and Neck Surgery, Chang Gung Memorial Hospital, Taoyuan, Taiwan; 9 Molecular Medicine Research Center, Chang Gung University, Taoyuan, Taiwan; 10 Research Center for Emerging Viral Infections, College of Medicine, Chang Gung University, Taoyuan, Taiwan

**Keywords:** Oral cavity squamous cell carcinoma, Autoantibodies, Biomarker, Machine learning

## Abstract

**Introduction:**

The incidence of oral cavity squamous cell carcinoma (OSCC) continues to rise. OSCC is associated with a low average survival rate, and most patients have a poor disease prognosis because of delayed diagnosis. We used machine learning techniques to predict high-risk cases of OSCC by using salivary autoantibody levels and demographic and behavioral data.

**Methods:**

We collected the salivary samples of patients recruited from a teaching hospital between September 2008 and December 2012. Ten salivary autoantibodies, sex, age, smoking, alcohol consumption, and betel nut chewing were used to build prediction models for identifying patients with a high risk of OSCC. The machine learning algorithms applied in the study were logistic regression, random forest, support vector machine with the radial basis function kernel, eXtreme Gradient Boosting (XGBoost), and a stacking model. We evaluated the performance of the models by using the area under the receiver operating characteristic curve (AUC), with simulations conducted 100 times.

**Results:**

A total of 337 participants were enrolled in this study. The best predictive model was constructed using a stacking algorithm with original forms of age and logarithmic levels of autoantibodies (AUC = 0.795 ± 0.055). Adding autoantibody levels as a data source significantly improved the prediction capability (from 0.698 ± 0.06 to 0.795 ± 0.055, *p* < 0.001).

**Conclusions:**

We successfully established a prediction model for high-risk cases of OSCC. This model can be applied clinically through an online calculator to provide additional personalized information for OSCC diagnosis, thereby reducing the disease morbidity and mortality rates.

**Supplementary Information:**

The online version contains supplementary material available at 10.1186/s12903-022-02607-2.

## Introduction

Oral cancer incidence is increasing globally, and this form of cancer is associated with a low average survival rate [1–3]. Developed countries have higher rates of oral cancer incidence, whereas less-developed countries have higher rates of disease mortality [4]. Oral cavity squamous cell carcinoma (OSCC) accounts for over 90% of oral cancer cases [5]. In more than 50% of patients, the OSCC diagnosis is delayed, and over half of patients are in an advanced stage (overall pathological stage III–IV) of the disease by the time of diagnosis [6]. In the past few decades, the effectiveness of OSCC detection has not improved considerably [7]. In Taiwan, over 40% of patients received a diagnosis of OSCC at a late stage [8], leading to poor disease prognosis and treatment failure [9, 10]. Therefore, an accurate diagnosis of OSCC, especially at its early stage, is crucial to improve the survival rate [11].

Oral potentially malignant disorders (OPMDs) consist of premalignant lesions that often progress to OSCC [12, 13]. Currently, conventional oral examination (COE) is the classical method for premalignant epithelium and oral cancer detection; however, differentiating OPMD and lesions with no risk of cancer by using COE remains challenging [14]. People lack knowledge about OPMD symptoms, and even clinicians may miss the signs of OPMD [2]. Therefore, developing an OPMD diagnostic tool to complement the COE conducted by clinicians can help patients to receive appropriate treatments in time and can help prevent malignant transformation.

Autoantibodies are antibodies produced against substances formed by a person’s own body and are expressed at low concentrations in healthy cells and at abnormally high concentrations in tumor cells [15]. Autoantibodies are potential biomarkers of breast [16], lung [17], colon [18], head and neck [19], esophageal [20, 21], and prostate [22] cancers. Several autoantibodies have been reported as OSCC biomarker candidates [23, 24]. Each of these reported autoantibodies exhibits a limited sensitivity or specificity for detecting OSCC, and whether a combined panel of these autoantibodies would be more effective than a single autoantibody in diagnosing the disease remains uncertain. Therefore, further verifying the efficacy of using these biomarkers together with demographic and behavioral data to identify patients with a high risk of OSCC is necessary.

Machine learning is a branch of artificial intelligence (AI) that enables computers to learn from previous data and make predictions [25]. Machine learning techniques are beneficial and are commonly applied to several cancers for predicting diagnosis, recurrence, metastasis, and prognosis [26–30]. We used machine learning models to predict high-risk cases of OSCC by using salivary autoantibody levels and demographic and behavioral data and evaluated the efficacy of salivary biomarkers in detecting OSCC.

## Methods

### Study participants and design

Participants were recruited from a teaching hospital, namely Chi-Mei Medical Center (Liouying, Taiwan), from September 2008 to December 2012 [23]. Inclusion criteria were Taiwanese adults > 30 years of age with current or previous habitual behaviors, such as smoking or betel nut chewing, following the oral cancer screen project launched by the Health Promotion Administration, Taiwan. Participants received a visual oral cavity examination by a trained dentist or physician and were divided into high-risk (patients with OSCC and high-risk OPMD, as confirmed by biopsy) and low-risk (patients with low-risk OPMD and healthy) groups on the basis of the criteria described in a previous study [23]. Salivary samples were collected at the time of recruitment, and saliva processing and autoantibody detection were performed in 2018. The experimental procedure of autoantibody detection was described in a previous study [23]. Before collection of saliva specimens, volunteers avoided smoking, eating, and drinking for at least 2 h. To remove cell debris, collected saliva samples were centrifuged at 3000×*g* for 15 min at 4 °C. The supernatants were immediately treated with a mixture of protease inhibitors (2 μL/mL; Cat. No. P8340, Sigma-Aldrich, Burlington, MA, USA), aliquoted into a volume of 100 μL, and then stored at a − 80 °C freezer. To avoid protein aggregation and degradation, saliva samples with more than one freeze–thaw cycle were not used. Using the strict protocol of collection and storage, the property and quality of saliva samples were preserved [24]. We evaluated the salivary autoantibody levels and selected 9 oral cancer-related proteins, ANXA2, CA2, HSPA5, ISG15, KNG1, MMP1, MMP3, PRDX2, SPARC, identified in a previous study [23] and p53 as the biomarker candidates of OSCC. Demographic and behavioral data, including sex, age, smoking, alcohol consumption, and betel nut chewing, were obtained. All participants signed the informed consent form before undergoing screening and treatment. The study was approved by the Institutional Review Board of Chi-Mei Medical Center (No. 10012-L02). Model reporting followed the TRIPOD (transparent reporting of a multivariable prediction model for individual prediction or diagnosis) guidelines [31].

### Data preprocessing for model development

**Fig. 1:**
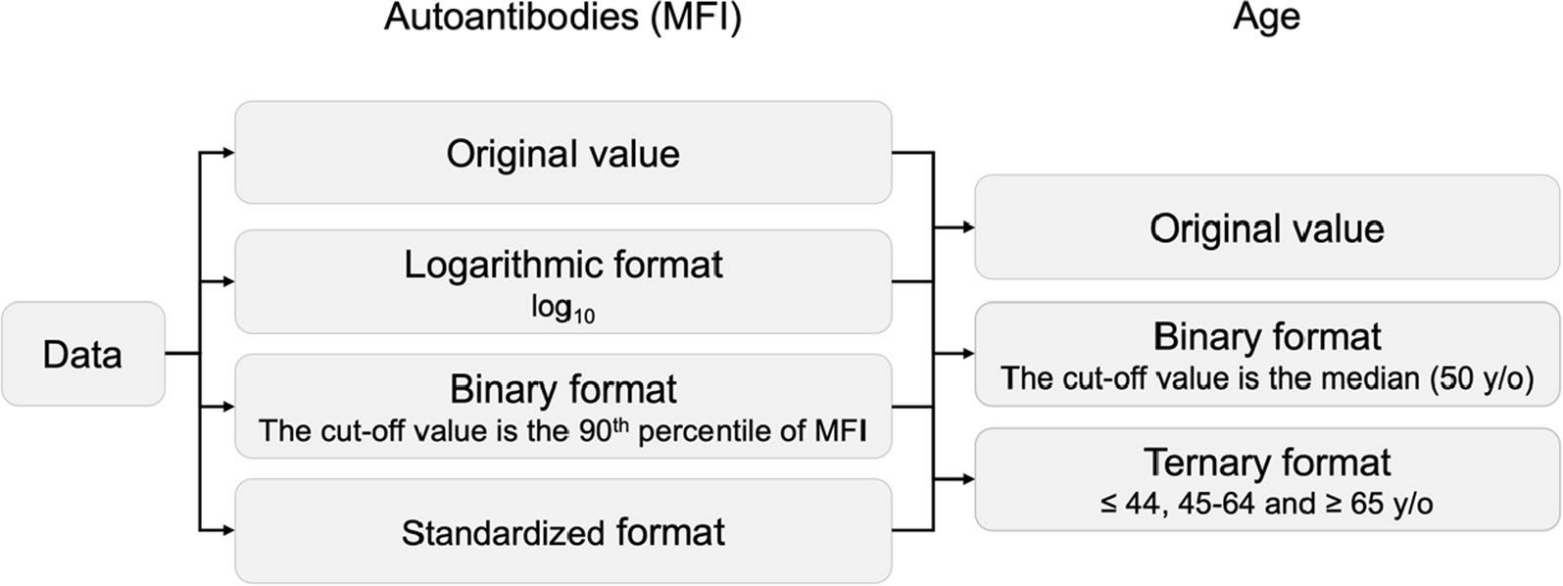
Flow chart of median fluorescent intensity (MFI) and age variable processing

For continuous variables, namely age and the mean fluorescence intensity (MFI) values of autoantibodies, we used the original values to develop prediction models. Moreover, we converted the MFI value into a binary, logarithmic, or standardized format and age into a binary or ternary format to evaluate the effect of using different forms of data (Fig. 1). In the training set, the 90^th^ percentile of the MFI value was set as the cutoff value for transforming the MFI value into the binary format. The median age (50 years) was set as the cutoff value for transforming age into the binary format; for transforming age into the ternary format, the cutoff values were set as ≤ 44, 45–64, and ≥ 65 years, based on the risk of oral cancer [32]. There were no missing values in the dataset.

### Development of prediction models

**Fig. 2:**
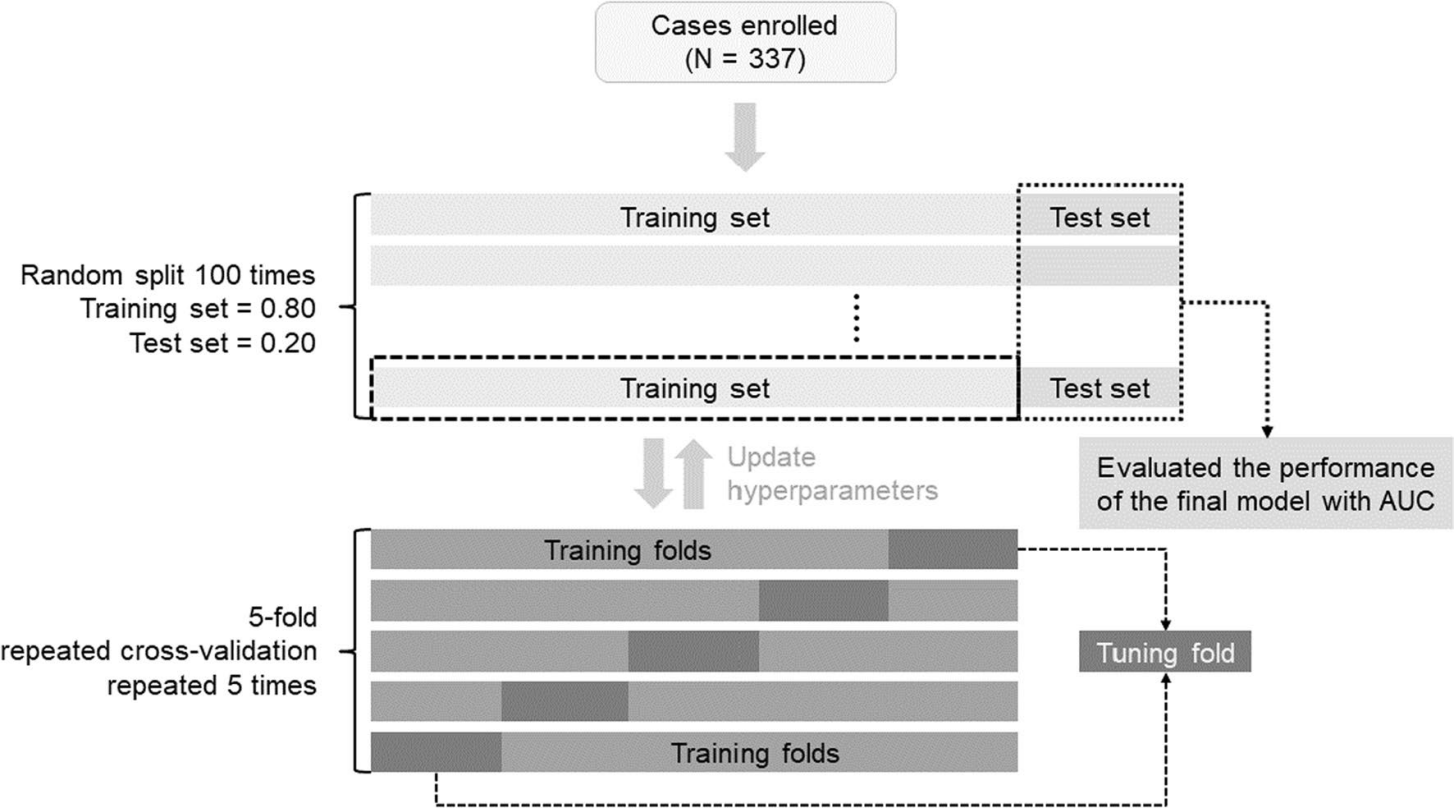
Flow chart of prediction model development and evaluation

We employed logistic regression (LR), random forest (RF), support vector machine (SVM) with the radial basis function (RBF) kernel, eXtreme Gradient Boosting (XGBoost), and a stacking model containing all the aforementioned models (i.e., LR, RF, SVM, and XGBoost) to develop the risk prediction models for OSCC. We performed a five-fold cross-validation that was repeated five times to optimize the model parameters during each round of model development (Fig. 2). All tuning parameters and the range of tuning for model development are listed in Additional file 1: Table S1.

LR is a statistical model that uses a logistic function to model a binary dependent variable [33]. An SVM with an RBF kernel constructs a classification model for a two-group issue; the data from two groups are separated using a hyperplane and transformed into a higher dimension [34]. RF is a bagging ensemble algorithm that uses random feature selection and consists of multiple classification trees [35]. XGBoost is an ensemble algorithm that optimizes distributed libraries under the gradient boosting framework and can predict a model from weak prediction models, usually decision trees. Thus, XGBoost uses a regularized model to control overfitting, and this gives it better performance [36]. Model stacking is an ensemble method that combines the prediction results of other models (LR, RF, SVM, and XGBoost) to generate a new ensemble model. Stacking combination was performed using lasso regression, a linear regression that uses the L1 penalty for both fitting and penalization of coefficients. Lasso regression performs both feature selection and regularization to improve prediction accuracy and model interpretability [37]. The caret [38], tidymodels [39], and stacks [40] packages in R software (R Core Team, Vienna, Austria) were used to develop and evaluate the predictive models.

### Evaluation of prediction models

The machine learning models for distinguishing high-risk and low-risk groups were trained with 80% of the data and tested using the remaining 20% of the data. To better estimate the performance of prediction models, we randomly split the training–test dataset 100 times to construct and evaluate 100 models for each algorithm (Fig. 2). All prediction models were then evaluated using the test sets. The area under the receiver operating characteristic curve (AUC) was used to evaluate model performance. The optimal model was used to build an online calculator. We applied the *International Journal of Medical Informatics* (IJMEDI) checklist for medical AI assessment (Additional file 1: Table S2) [41].

### Effectiveness of autoantibodies as biomarkers for OSCC risk prediction

Variable selection was performed using the permutation-based variable importance measure, which is based on the hypothesis that if a variable is important, the model’s performance will worsen after permuting the values of the variable. The larger the change in performance is, the more important is the variable. We used 1 − AUC as the loss metric; the higher the value of this metric is, the higher is its importance as a predictive variable [42]. The DALEX [42] and DALEXtra [43] packages were used to explain the model and evaluate the variable importance.

To evaluate the effectiveness of autoantibodies and demographic and behavioral data in identifying patients with high OSCC risk, we compared the performance of prediction models that used both autoantibodies and demographic and behavioral data with those that used patient characteristics alone.

### Statistical analysis

Continuous variables are expressed as medians (interquartile range) for skewed distributions and were analyzed using the Mann–Whitney U test to determine the differences between the two groups. Categorical variables are presented as percentages and were calculated using the chi-square or Fisher’s exact test. The scale function, converting each original value into a z-score, in R was used for standardization. The AUC values of prediction models, from the 100 times of performance evaluations, were compared using repeated-measures ANOVA (rANOVA), and the Holm–Bonferroni post hoc test was used to compare processing strategies and algorithms. All statistical tests were two-sided, and statistical significance was defined as *p* < 0.05. Statistical analyses were performed using R 4.1.2.

## Results

### Patient characteristics and salivary IgA autoantibody levels

A total of 337 participants were enrolled in this study. The baseline characteristics and the salivary levels of 10 autoantibodies are listed in Table 1. Among participants, 331 (98.2%) were male, 306 (90.8%) smoked, 118 (35.0%) consumed alcohol, and 271 (85.4%) chewed betel nut; the median age was 50.4 years (IQR = 12). Participants were stratified into high-risk (n = 190; 107 OSCC cases and 83 high-risk OPMD cases) and low-risk (n = 147; 55 low-risk OPMD cases and 92 healthy individuals) groups. Participants in the high-risk group were older (51.8 vs. 49.2 years, *p* = 0.003), consumed more alcohol (42.6% vs. 25.2%, *p* = 0.001), chewed more betel nut (92.1% vs. 65.3%, *p* < 0.001), and exhibited higher levels of all salivary autoantibodies (*p* < 0.005) except for anti-ANXA2 (*p* = 0.253).

**Table 1:** Patient characteristics and salivary autoantibody levels

	Overall	High-risk	Low-risk	*p*-value
No. of cases (%)	337	190 (56.4)	147 (43.6)	–
Sex, male, n (%)	331 (98.2)	188 (98.9)	143 (97.3)	0.41^a^
Age, range	31–82	32–82	31–78	0.003^b^
Years, median (IQR)	50.4 (17)	51.8 (15)	49.2 (19)	
Smoking, n (%)	306 (90.8)	176 (92.6)	130 (88.4)	0.258^c^
Alcohol consumption, n (%)	118 (35.0)	81 (42.6)	37 (25.2)	0.001^c^
Betel nut chewing, n (%)	271 (80.4)	175 (92.1)	96 (65.3)	< 0.001^c^
Autoantibodies, MFI, median (IQR)				
Anti-ANXA2	2727.2 (2611.4)	2791.8 (3079.5)	2600.1 (2244.6)	0.253^b^
Anti-CA2	1191.2 (1232.8)	1446.0 (1509.2)	1020.0 (947.3)	< 0.001^b^
Anti-HSPA5	607.8 (722.8)	757.5 (892.1)	457.1 (484.0)	< 0.001^b^
Anti-ISG15	1273.7 (1299.0)	1506.0 (1635.8)	1021.4 (1004.4)	< 0.001^b^
Anti-KNG1	4176.5 (4720.2)	4814.0 (6782.6)	3379.6 (2981.0)	< 0.001^b^
Anti-MMP1	1426.3 (1214.5)	1647.8 (1523.4)	1239.3 (953.8)	< 0.001^b^
Anti-MMP3	3907.2 (548.4)	4018.6 (777.1)	3828.9 (405.2)	< 0.001^b^
Anti-p53	1616.7 (1782.1)	1814.1 (2432.3)	1485.1 (1437.4)	0.003^b^
Anti-PRDX2	1038.0 (1286.3)	1336.4 (1525.1)	813.0 (865.5)	< 0.001^b^
Anti-SPARC	707.0 (724.8)	833.9 (956.5)	555.2 (507.2)	< 0.001^b^

*IQR* interquartile range, *MFI* median fluorescence intensity

*p-*values of high-risk and low-risk groups were compared

^a^Fisher’s exact test

^b^Mann–Whitney U test

^c^Chi-square test

### Comparison of prediction model performances

**Fig. 3:**
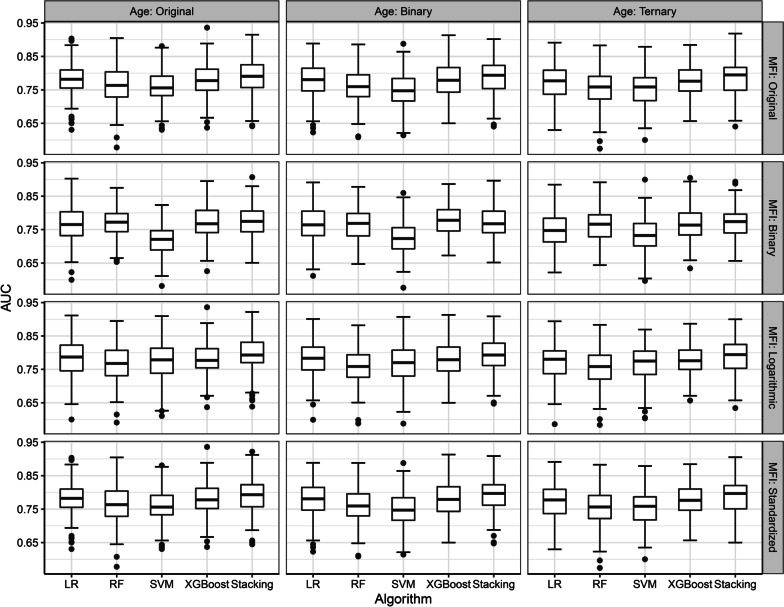
Performance of risk prediction models by using 12 data processing strategies. LR: logistic regression; RF: random forest; SVM: support vector machine with a radial basis function kernel; XGBoost: eXtreme Gradient Boosting

First, we evaluated different data processing strategies and five machine learning models for distinguishing high-risk from low-risk patients; the AUCs, from the test sets, are listed in Fig. 3 and Additional file 1: Table S3. The AUCs from the training sets are listed in Additional file 1: Table S4. The optimal machine learning algorithm for predicting high-risk OSCC cases, based on the AUC from the test sets, was the stacking method; rANOVA and post hoc analysis revealed significant differences among other machine learning algorithms (*p* < 0.001, Table 2). When building a model with the stacking method, the best data processing strategy was to use age in the original format and logarithmic autoantibody levels (AUC = 0.795 ± 0.055). Compared with transforming autoantibody levels to the binary format, transforming autoantibody levels to the logarithmic format resulted in significant improvement in prediction performance (*p* < 0.05, Additional file 1: Table S5). However, no significant difference in prediction performance was observed between transforming autoantibody levels to the logarithmic format and transforming autoantibody levels to the standardized format.

**Table 2:** Holm–Bonferroni post hoc test across machine learning models performed using age in the original format and logarithmic autoantibody levels

Algorithm (AUC)	LR 0.784 ± 0.057	RF 0.764 ± 0.057	SVM 0.772 ± 0.059	XGBoost 0.778 ± 0.051	Stacking 0.795 ± 0.055
RF	< 0.001	–	–	–	–
SVM	0.006	0.188	–	–	–
XGBoost	0.270	0.002	0.270	–	–
Stacking	0.001	< 0.001	< 0.001	< 0.001	–

*LR* logistic regression, *RF* random forest, *SVM* support vector machine with a radial basis function kernel, *XGBoost* eXtreme Gradient Boosting, *Stacking* a stacking model that contained all models, *SD* standard deviation

The calibration curves are provided in Additional file 1: Fig. S1, and the Brier scores were 0.125, 0.123, 0.151, 0.140, and 0.127 for the stacking, XGBoost, RF, SVM, and LR models, respectively. The lift chart is provided in Additional file 1: Fig. S2; the lift value in the highest risk group (top 5%) was 1.79 for all models, indicating that the positive predictive value in the highest risk group, as identified by the stacking model, was approximately two times higher than the average positive predictive value. The IJMEDI checklist is provided in Additional file 1: Table S2. An online calculator developed based on the optimal model is depicted in Additional file 1: Fig. S3.

### Autoantibodies as biomarkers for OSCC risk prediction

**Fig. 4:**
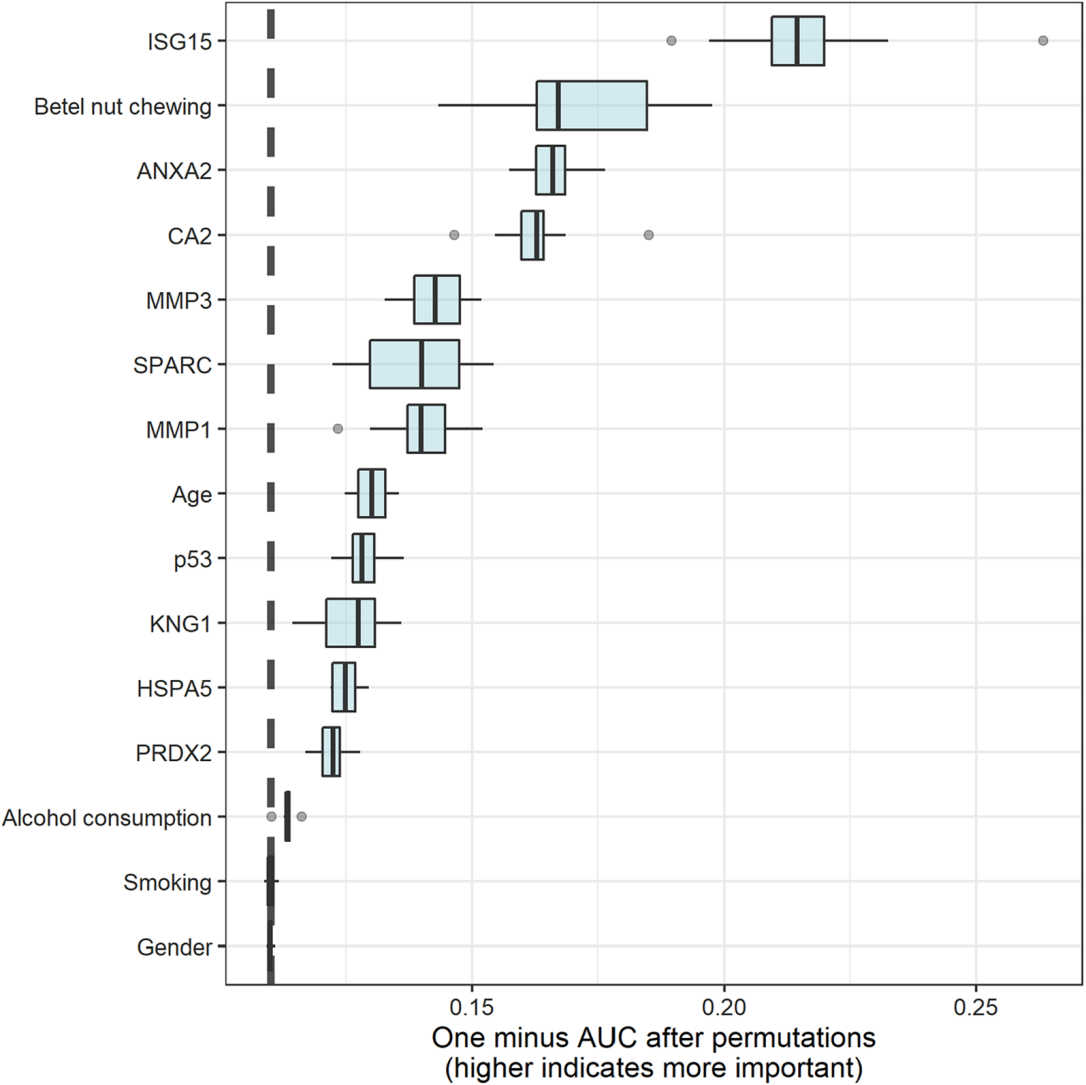
Variable importance in the stacking model

Variable importance, as identified by the stacking model, is provided in Fig. 4. Importance was calculated explicitly for each variable in the dataset, allowing variables to be ranked and compared with each other. Higher variable importance indicates that the variable contributes more to the AUC. The top five highest-ranking important variables in the stacking model were anti-ISG15, betel nut chewing, anti-ANXA2, anti-CA2, and anti-MMP3.

The AUC of the models developed through the stacking approach, using demographic and behavioral data together with age in the original format and logarithmic autoantibody levels, was 0.698 ± 0.060. Therefore, adding autoantibodies as biomarkers for identifying patients with a high risk of OCSS improved prediction performance by 13.9% (0.698 vs. 0.795).

## Discussion

We used machine learning models to successfully predict high-risk cases of OSCC and evaluated the efficiency of salivary autoantibody levels to detect patients with a high risk of OSCC accurately and reliably.

Oral visual inspection is the main method for evaluating the risk of OSCC; however, differentiating OPMD and lesions with no risk of cancer progression through oral inspection remains challenging for clinicians [14]. Compared with the levels of other biomarkers, autoantibody levels are usually steady; therefore, they are easily detected using reagents available in the market [44]. In addition, autoantibodies exhibit an enduring response to tumor-associated antigens [45]. Therefore, tumor-associated autoantibodies are clinically useful and can serve as biomarkers for OSCC screening. We demonstrated that evaluating salivary autoantibody levels can help physicians identify the risk of OSCC early. Our results revealed that the salivary levels of CA2 [46], HSPA5 [47], ISG15 [48], KNG1 [49], MMP1 [50], MMP3 [51, 52], p53 [53], PRDX2 [54], and SPARC [55] were significantly elevated in the high-risk group compared with the low-risk group, a result similar to those of previous studies (Table 1). The data revealed that the elevated salivary autoantibody levels were related to OSCC progression. Moreover, four of the five most important variables in the stacking model were autoantibodies (Fig. 4). Thus, using salivary autoantibody levels to assist OSCC screening is a promising strategy.

Although salivary autoantibody levels exhibited strong performance in detecting high-risk OSCC cases, traditional risk factors, such as betel nut chewing, are still crucial for estimating the risk of OSCC (Fig. 4). In previous studies, the majority of patients with OSCC had habitual behaviors, such as betel nut chewing and drinking alcohol [56–58]. Adding salivary autoantibody levels to these traditional risk factors increased the model capacities.

The salivary samples were collected from 2008 to 2012, and the autoantibody detection was performed in 2018. Previous studies revealed a statistical decrease in concentration of salivary immunoglobulin A and hormones as storage time increased [59, 60]. However, before long-term storage, the salivary samples used in this study were centrifuged, treated with a protease inhibitor mixture, and stored at a − 80 °C freezer to avoid protein degradation. This strict protocol can ensure the quality of saliva samples [61]. More importantly, detection of salivary anti-p53 levels has been carried out in our previous study in 2014 [24]. Part of saliva samples used in 2014 is the same to that in the present study. For each identical patient or healthy control, salivary level of anti-p53 in the present study is similar to that acquired in 2014, indicating that the salivary properties might be appropriately preserved in terms of salivary immunoglobulin.

There are several limitations to this study. First, we recruited participants from a single institution in Taiwan; therefore, external validation is not available and our results may not be generalizable to other regions. Although we performed an internal evaluation with 100 randomly split training–test datasets to minimize model evaluation bias, multiple-center studies may be required to increase model generalizability. Second, we only included a small number of cases; therefore, future studies should include a large sample size collected from multiple centers. The application of autoantibodies as biomarkers should be validated by performing a cohort study to evaluate the efficiency of autoantibodies in diagnosing patients with early-stage OSCC. Finally, we evaluated the levels of only 10 salivary autoantibodies [23]; however, other potential salivary protein biomarkers should be applied to detect high-risk OSCC cases [49]. Moreover, other factors, such as human papillomavirus infection, ultraviolet light, poor nutrition, and genetic syndromes [62], may increase the risk of OSCC and should be included in future studies.

## Conclusion

We successfully established a prediction model for high-risk cases of OSCC. Combining the online calculator, which was developed on the basis of the proposed model, with a common clinical visual exam would help in the early diagnosis of OSCC, thereby reducing the disease morbidity and mortality rates.

## Supplementary Information

**Additional file 1.** The supporting information including Supplemental Tables S1–S5 and Supplemental Figures S1–S3.

## Data Availability

Authors' data use agreement for the dataset does not permit public posting of this patient information. The R codes for generating analysis results were available at: https://doi.org/10.57770/QTYQZS.

## Abbreviations

OSCC: Oral cavity squamous cell carcinoma
OPMDs: Oral potentially malignant disorder
COE: Conventional oral examination
MFI: Mean fluorescence intensity
LR: Logistic regression
RF: Random forest
SVM: Support vector machine
XGBoost: EXtreme Gradient Boosting
AUC: Area under the receiver operating characteristic curve
rANOVA: Repeated-measures ANOVA
